# *Staphylococcus aureus* Bacteremia Complicated by Psoas Abscess and Infective Endocarditis in a Patient with Atopic Dermatitis

**DOI:** 10.1155/2017/4920182

**Published:** 2017-12-10

**Authors:** Ichiro Tsuboi, Tetsuya Yumoto, Tatsuya Toyokawa, Katsunori Matsueda, Joichiro Horii, Hiromichi Naito, Atsunori Nakao

**Affiliations:** ^1^Department of Gastroenterology, National Hospital Organization Fukuyama Medical Center, 4-14-17, Okinogamicho, Fukuyama-shi, Hiroshima 720-8520, Japan; ^2^Advanced Emergency and Critical Care Medical Center, Okayama University Hospital, 2-5-1 Kita-ku, Shikata-cho, Okayama-shi, Okayama 700-8558, Japan

## Abstract

The close relationship between atopic dermatitis (AD) and infective endocarditis (IE) has been implicated. *Staphylococcus aureus* colonization is frequently seen observed in AD patients' skin lesions. Although a case of IE due to *S. aureus* bacteremia in an AD patient has been sporadically reported, a case of *S. aureus* bacteremia complicated by psoas abscess and IE has not been previously reported. A 42-year-old man with a history of AD presented to our hospital complaining of fever, fatigue, chills, lower right back pain, and poor appetite for a week. His blood cultures showed growth of *S. aureus*. On day 3, the patient presented acute cardiac failure and was diagnosed with IE based on echocardiogram examination. Since the patient's cardiac failure did not respond to medication, an emergency surgery was performed on the fourth day of hospitalization. The patient underwent successful surgical treatment of the heart lesions and subsequent percutaneous drainage of psoas abscess and received intensive antibiotics, which successfully improved his condition. Our report emphasizes awareness of the association between AD and invasive *S. aureus* infections.

## 1. Introduction

Atopic dermatitis (AD), an ongoing, relapsing, eczematous skin disorder marked by inflammation and pruritus, is caused by various specific allergens and unspecific stimuli. Being one of the most prevalent skin diseases, AD affects up to 1–3% of adults and 20% of children and in most nations all over the world [1].

Patients with AD are more vulnerable to infection due to impaired skin barrier. *Staphylococcus aureus,* in particular, is frequently seen in AD patients' skin lesions [2]. An association between severe AD and acute bacterial endocarditis after recurring staphylococcal skin infection was first reported by Pike and Warner [3]. Since then, a close relationship between AD and infective endocarditis (IE) has been advocated [4].

Although a substantial number of case reports have depicted the simultaneous presence of IE and AD, *S. aureus* bacteremia complicated with both psoas abscess and IE has not been previously described in a patient with AD [5–7]. Herein, we report a case of psoas abscess and IE caused by *S. aureus* in a patient with AD. Sharing our experience with readers may help clinicians develop a proper and early diagnostic strategy, a key to the successful management of invasive *S. aureus* infections in AD patients.

## 2. Case Presentation

A 42-year-old man with a history of AD presented to our hospital. His AD skin lesion was treated with topical corticosteroids (diflucortolone valerate). He denied any scratches or recent skin injuries and had never used intravenous drugs. He complained of fever, fatigue, chills, lower right back pain, and poor appetite for a week. He had no previous history of heart disease or rheumatic fever. On physical examination, his body temperature was 38.3°C, pulse rate was 84/min, blood pressure was 106/76 mmHg, and respiratory rate was 18 breaths/min. His neurologic examination was unremarkable; he had a Glasgow Coma Scale score of 15 without altered sensorium, meningeal signs, or focal deficits. He had no conjunctival pallor or icterus or jugular vein distention. Cardiovascular and respiratory exam showed that his murmur and breath sounds were normal.

**Figure 1:**
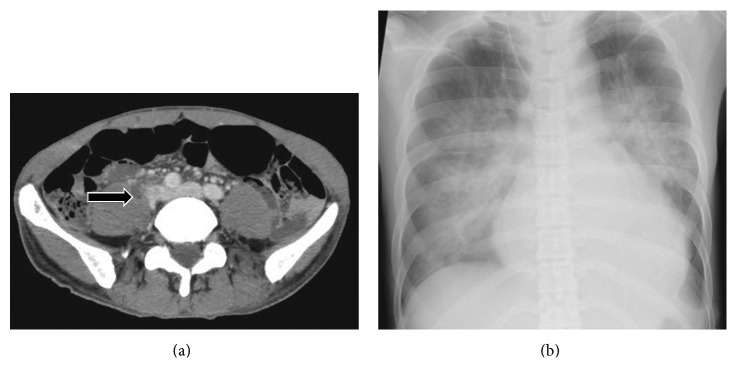
(a) There was a low-density foci with enhancement of the wall in the patient's right iliopsoas muscle, raising suspicion of iliopsoas abscess. (b) The result of chest radiography on day 3 showed severe cardiomegaly, measured as a cardiothoracic ratio of 61% with pulmonary edema.

A skin survey demonstrated no signs such as Osler's node or Janeway lesion. Laboratory tests indicated a white blood cell count of 3.5 × 10^3^/µl, platelet count of 98 × 10^3^/µl, and C-reactive protein level of 37.29 mg/dl. His electrocardiogram and chest X-ray were unremarkable, without an enlarged heart shadow or a consolidation. Contrast-enhanced computed tomography (CT) revealed a low-density mass in the iliopsoas muscle, raising suspicion of an iliopsoas abscess (Figure 1(a)). Two sets of blood cultures were obtained. Based on the preliminary clinical diagnosis of systemic inflammatory response associated with primary psoas abscess, empirical administration of intravenous meropenem was initiated at 1 gram every eight hours.

**Figure 2:**
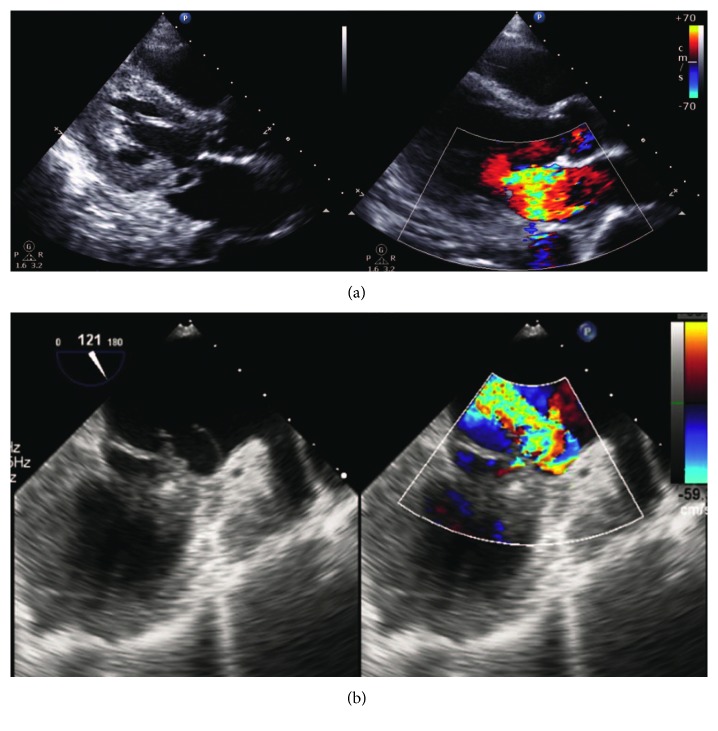
(a) In parasternal long-axis view, wall motion was normokinetic and ejection fraction was over 60%. Posterior leaflet of mitral valve (P1) had a valvular aneurysm and perforation of the valvular aneurysm. Severe mitral regurgitation is shown in color Doppler echo imaging. (b) Transesophageal echocardiography showed that posterior leaflet of mitral valve (P1) had a valvular aneurysm, with perforation of the valvular aneurysm and severe mitral regurgitation, but did not have vegetation or abscess of the mitral valve annulus.

The day following admission, the patient complained of chest discomfort and continued fever. Electrocardiogram and chest X-ray did not demonstrate any abnormal findings. The blood cultures exhibited growth of *S. aureus* sensitive to vancomycin; the antimicrobial treatment was then switched from meropenem to vancomycin at 1 gram every 12 hours. Three days after admission, his blood pressure and heart rate were 100/76 mmHg and 160/min, respectively, with blood lactate levels of 1.1 mmol/L. Fluid resuscitation and inotropic support to maintain circulation were initiated. Chest X-ray revealed cardiomegaly with pulmonary edema (Figure 1(b)). Due to increasing chest discomfort, the patient was transferred to the intensive care unit and required mechanical ventilation and endotracheal intubation with positive airway pressure. Transthoracic echocardiography in the parasternal long-axis view revealed normal wall motion and fair ejection fraction > 60%. The posterior leaflet of the mitral valve had a valvular aneurysm with perforation. Color Doppler echocardiography revealed severe mitral regurgitation (Figure 2(a)). Cardiomegaly was observed due to enlargement of left atrium and left ventricle, although it was uncommon in the setting of acute mitral regurgitation. Subsequent transesophageal echocardiography notably failed to show any obvious vegetation or abscess of the mitral valve annulus (Figure 2(b)). As these results met both the modified Duke Criteria and the Jones criteria, we made the diagnosis of IE. Since conservative medical management, including antibiotic administration, was suboptimal for curative treatment, we concluded that valvular surgery was required. The patient was transferred to the cardiovascular surgery department and underwent emergency surgery on the third day after admission. Surgery revealed the presence of vegetations on the destroyed left cusps, and the infection had reached the annulus. The anterior commissure leaflet was resected together with the vegetation and ruptured chordae tendineae, followed by mitral valve replacement.

CT-guided percutaneous drainage of the abscess in the right psoas muscle was successfully carried out two weeks after the surgery. The antibiotic treatment with vancomycin in combination with ceftriaxone was continued for four weeks, followed by sole antibiotic therapy with ceftriaxone, which was discontinued after three consecutive blood cultures had been confirmed negative. The postoperative course was unremarkable, and the patient was discharged from the institution 45 days later.

## 3. Discussion

This is the first report of *S. aureus* bacteremia complicated with both psoas abscess and IE in a patient with AD. Our report highlights the fact that AD can predispose to development of invasive *S. aureus* infections including psoas abscess and IE.

AD is the most common persistent inflammatory skin disorder characterized by intense pruritus and recurrent erythema. Patients with AD exhibit significant skin barrier disruption which leads to increased susceptibility to infection [8]. The human skin innate immune system includes the antimicrobial peptides β-defensins (HBD-2) and cathelicidins (LL-37), which are induced by inflammation and have antimicrobial, antifungal, and antiviral properties. These peptides are deficient in AD patients, resulting in increased susceptibility to microorganisms and providing an inviting environment for invasion, proliferation, and colonization of *S. aureus* [9, 10].

As skin colonization with *S. aureus* is universal in AD patients, invasive *S. aureus* infections including IE occasionally occur in patients with AD [11]. As for IE, *S. aureus* is the most common microorganism responsible for IE. Risk factors of IE due to *S. aureus* include prosthetic heart valve, injecting drug use, and persistent bacteremia [12]. Although there is no clear evidence of a higher frequency of the coexistence of IE and AD, IE should be known as an important complication in this familiar skin disorder [4]. Higher C-reactive protein and persistent fever in the setting of *S. aureus* bacteremia are associated with IE as the present case showed [12, 13].

IE due to *S. aureus* more frequently causes complications including septic embolization and metastatic infection than other pathogens [14]. Horino et al. described that metastatic infections such as psoas abscess, spondylitis, IE, pulmonary abscess, and epidural abscess occurred in 14 of 73 patients with *S. aureus* bacteremia [13]. From the aspect of psoas abscess, since it can present with IE, patients with psoas abscess should be investigated for IE particularly caused by *S. aureus* [15]. To our knowledge, this is the first case of psoas abscess and IE due to *S. aureus* bacteremia in a patient with AD and otherwise healthy. Invasive *S. aureus* infections including IE and metastatic abscess should be considered in AD patients presenting with prolonged fever or fatigue.

## 4. Conclusion

AD can be a potential risk factor for invasive *S. aureus* infections including IE and psoas abscess. Physicians should be aware of the association between AD and invasive *S. aureus* infections to develop a proper and early diagnostic strategy.
